# Transcriptome Analysis of Cells Exposed to Actinomycin D and Nutlin-3a Reveals New Candidate p53-Target Genes and Indicates That CHIR-98014 Is an Important Inhibitor of p53 Activity

**DOI:** 10.3390/ijms222011072

**Published:** 2021-10-14

**Authors:** Barbara Łasut-Szyszka, Beata Małachowska, Agnieszka Gdowicz-Kłosok, Małgorzata Krześniak, Magdalena Głowala-Kosińska, Artur Zajkowicz, Marek Rusin

**Affiliations:** 1 Center for Translational Research and Molecular Biology of Cancer, Maria Skłodowska-Curie National Research Institute of Oncology, 44-101 Gliwice, Poland; Barbara.Lasut-Szyszka@io.gliwice.pl (B.Ł.-S.); Agnieszka.Gdowicz-Klosok@io.gliwice.pl (A.G.-K.); Malgorzata.Krzesniak@io.gliwice.pl (M.K.); Artur.Zajkowicz@io.gliwice.pl (A.Z.); 2 Department of Radiation Oncology, Albert Einstein College of Medicine, Bronx, NY 10461, USA; beata.malachowska@einsteinmed.org; 3 Department of Bone Marrow Transplantation and Oncohematology, Maria Skłodowska-Curie National Research Institute of Oncology, 44-101 Gliwice, Poland; Magdalena.Glowala-Kosinska@io.gliwice.pl

**Keywords:** p53, innate immunity, GSK-3, RNA-Seq, kinase inhibitor

## Abstract

Co-treatment with actinomycin D and nutlin-3a (A + N) strongly activates p53. Previously we reported that CHIR-98014 (GSK-3 kinase inhibitor), acting in cells exposed to A + N, prevents activation of *TREM2*-an innate immunity and p53-regulated gene associated with Alzheimer’s disease. In order to find novel candidate p53-target genes and genes regulated by CHIR-98014, we performed RNA-Seq of control A549 cells and the cells exposed to A + N, A + N with CHIR-98014 or to CHIR-98014. We validated the data for selected genes using RT-PCR and/or Western blotting. Using CRISPR/Cas9 technology we generated p53-deficient cells. These tools enabled us to identify dozens of candidate p53-regulated genes. We confirmed that p53 participates in upregulation of *BLNK*, *APOE* and *IRF1*. *BLNK* assists in activation of immune cells, *APOE* codes for apolipoprotein associated with Alzheimer’s disease and *IRF1* is activated by interferon gamma and regulates expression of antiviral genes. CHIR-98014 prevented or inhibited the upregulation of a fraction of genes stimulated by A + N. Downregulation of GSK-3 did not mimic the activity of CHIR-98014. Our data generate the hypothesis, that an unidentified kinase inhibited by CHIR-98014, participates in modification of p53 and enables it to activate a subset of its target genes, e.g., the ones associated with innate immunity.

## 1. Introduction

The tumor suppressor protein p53 coded by *TP53* gene is activated by stress factors acting under natural conditions, e.g., UV radiation, oxidative stress, oncogene activation. However, the protein is also activated by stress factors, associated with anticancer therapy, e.g., high-dose ionizing radiation, chemotherapeutic DNA-damaging agents. These p53 activators are not found in the natural environment but they exploit the ability of p53 to induce cell cycle arrest or apoptosis. There are also experimental anticancer drugs (e.g., nutlin-3a), which were designed specifically to activate p53 by antagonizing MDM2 protein, which is negative regulator of p53 [1].

We found that two anticancer agents actinomycin D and nutlin-3a acting together (A + N) synergize in activation of p53 protein and consequently, they synergize in stimulation of p53-regulated genes [2]. The strong activation of p53 by these agents allowed to identify genes, whose regulation by p53 was not observed before, e.g., the genes coding for proteins involved in innate immunity: *TREM2*, *TMEM173* (*STING1*), *NLRP1*, *NLRX1*, *SOCS1* [3,4]. The synergy between actinomycin D and nutlin-3a in activation of some genes is exceptionally strong. This group includes *CASP1* and *H19* as well as the aforementioned *TREM2* and *STING1* [3,4,5]. *CASP1* codes for caspase-1, an innate immunity protein, which induces programmed cell death called pyroptosis associated with secretion of pro-inflammatory cytokines-interleukin-1β and interleukin-18 [6]. *H19* gene directs the production of long non-coding RNA molecules with anti-apoptotic activity [7].

Unexpectedly, we found that in A549 lung cancer cell line the drug combination A + N induced the expression of several elements of a signaling pathway encompassing TREM2 (a receptor), TYROBP (a co-receptor), SYK (a kinase) and BLNK (an adaptor protein) [3]. This signaling pathway is found principally in cells involved in innate immunity, e.g., microglial cells [8], hence, its appearance in lung cancer cells exposed to A + N was very surprising. Interestingly, the induction of all these proteins was significantly attenuated by sub-micromolar concentration of CHIR-98014 (in the following part abbreviated to CHIR), which is considered as a specific inhibitor of glycogen synthase kinase 3, α and β isoforms coded by *GSK3A* and *GSK3B* genes, respectively [9,10]. This observation suggested that GSK-3 is indispensable for activation of p53 and stimulation of some of its target genes. This enzyme, not only regulates the synthesis of glycogen, but also regulates the plethora of other cellular processes, hence its name may be misleading suggesting exclusive role in cellular metabolism. GSK-3 influences embryonic development, neuronal function, cell proliferation and differentiation, apoptosis, inflammation and immune response [10]. The p53 can be activated by phosphorylation catalyzed by GSK-3β [11], hence our selection of CHIR to test the role of this kinase in activation of p53 triggered by A + N treatment.

In the current project we decided to perform global search for genes regulated by A + N as well as by CHIR. We started from transcriptome analysis of cells in control conditions or exposed to A + N, to A + N + CHIR or to CHIR alone. In this way we identified dozens of candidate p53-target genes and found what genes upregulated by A + N are additionally modulated by GSK-3 kinase inhibitor. Based on RNA-Seq data and on experiments with engineered p53-deficient cells, we found that p53 participates in upregulation of two genes-*APOE* and *BLNK*, coding for elements of TREM2 signaling pathway. Analysis of RNA-Seq results revealed that there are at least two groups of genes as far as CHIR is considered. One group consists of genes strongly upregulated by A + N, whose activation is substantially reduced by CHIR. The other group consists of genes, whose activation by A + N is not influenced by the inhibitor. Because the first group contains anti-apoptotic genes stimulated by p53 (e.g., *CRYAB*, *H19*) and the second group contains many well-known p53-activated pro-apoptotic genes (e.g., *PMAIP*, *BBC3*), we hypothesized that CHIR promotes apoptosis because it prevents expression of anti-apoptotic genes and does not influence the expression of pro-apoptotic ones. Biologically, the hypothesis was positively tested, i.e., CHIR promoted apoptosis in cells exposed to A + N. However, the molecular mechanism of the induction of apoptosis is more complicated than expected. The induction of pro-apoptotic caspase-3 by A + N + CHIR was visible also in p53-null cells and CHIR induced the expression of mRNAs coding for GDF15 or BCL2L15 pro-apoptotic proteins. Moreover, downregulation of GSK-3 did not mimic CHIR activity, suggesting that CHIR can act independently from inhibiting GSK-3. Thus, our data generate the hypothesis that CHIR inhibits a kinase, which participates in activation of p53 and upregulation of a subset of its target genes.

We selected A549 lung adenocarcinoma cell line for this study because it is a frequently used model of lung cancer with wild-type *TP53* employed by us earlier [2,3,4,5]. Moreover, to the best of our knowledge, the transcriptomic data on p53-target genes in A549 cell line were not published [12]. Unexpectedly, in the course of our recent research we found that in cancer cells, strongly activated p53 was able to induce expression of many genes, which were known to be expressed only in immune cells [3,4]. Studying the regulation of immune-related genes in lung cancer cells may yield new valuable information concerning the quickly expanding field of immunotherapy of cancer.

## 2. Results

### 2.1. Results of Transcriptome Sequencing

The primary purpose of the transcriptomic analysis was to screen for genes upregulated by A + N and to identify genes activated by A + N and modulated by CHIR. The data (fastq files) are deposited in the Sequence Read Archive under the accession number PRJNA757776. The results for the selected genes were validated by RT-PCR performed on RNA samples isolated from three biological repeats. The results for other genes were validated by Western blot.

The pair-wise comparisons of gene expression measured by transcriptome sequencing are shown in Supplementary Tables S2–S4. We compared every treatment modality, i.e., A + N, A + N + CHIR and CHIR against control cells (mock-treated). The raw data used to generate these tables are available upon request from the first or corresponding author.

**Figure 1:**
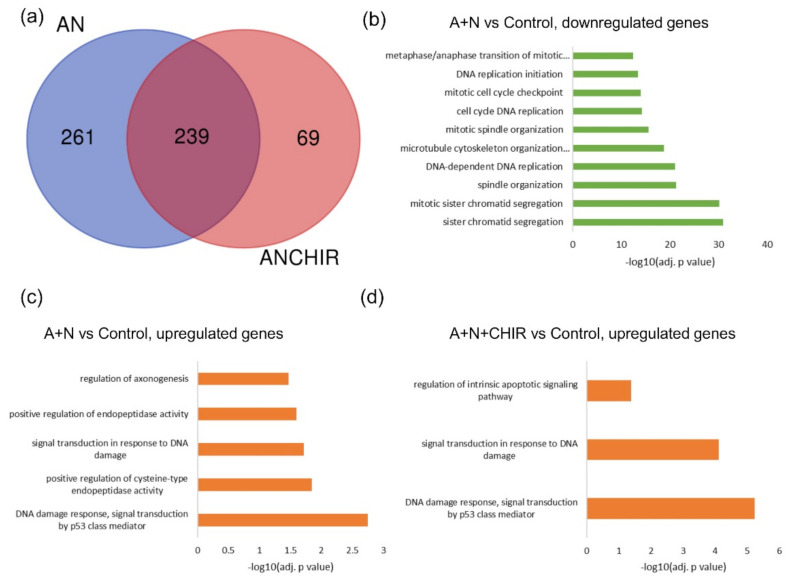
Results of RNA-seq analysis of actinomycin D (A), nutlin-3a (N) and CHIR treatment combination on lung adenocarcinoma cells (A549). (**a**) Venn diagram comparing lists of genes significantly upregulated in response to treatment with AN and with AN in the presence of CHIR-98014 (ANCHIR). (**b**–**d**) Top 10 (if available) overrepresented pathways with adjusted *p* value < 0.05, size < 200 and intersection to query size ratio >0.03 for: (**b**) down-regulated genes in A + N versus control comparison, (**c**) up-regulated genes in A + N versus control comparison and (**d**) up-regulated genes in A + N + CHIR versus control comparison (*n* = 1).

To estimate globally how CHIR treatment influences gene expression, we compared two groups of genes-significantly upregulated by A + N and significantly upregulated by A + N + CHIR treatment. The results of this analysis are presented on Venn diagram (Figure 1a). Generally, the expression of half of upregulated genes in cells exposed to A + N was also significantly upregulated in conditions with added CHIR (239), expression of another half of them was not significantly up-regulated in conditions with added CHIR (probably inhibited by CHIR) (261), whereas the expression of 69 genes was only significantly up-regulated in conditions with added CHIR. Thus, we concluded that in cells exposed to A + N, CHIR induces vast changes in the transcriptome.

Next, we employed enrichment analysis for genes significantly up- or downregulated by A + N. Not surprisingly, GO terms associated with DNA replication initiation, mitotic spindle organization, DNA-dependent DNA replication were enriched in the list of genes significantly downregulated by A + N treatment (Figure 1b). These substances potently arrest the cell cycle progression at G1 or G2 [2]. The GO term the most significantly enriched in the group of genes upregulated by A + N is “DNA damage response, signal transduction by p53 class mediator” (Figure 1c). The enrichment analysis based on GO terms also indicated significant enrichment of genes belonging to the term “Regulation of axonogenesis”. Interestingly, we noticed that in the data from comparison between control cells and cells exposed to A + N + CHIR, a GO term “Regulation of intrinsic apoptosis signaling pathway” was significantly enriched, what suggested that CHIR may modulate sensitivity of cells to apoptosis (Figure 1d).

**Figure 2:**
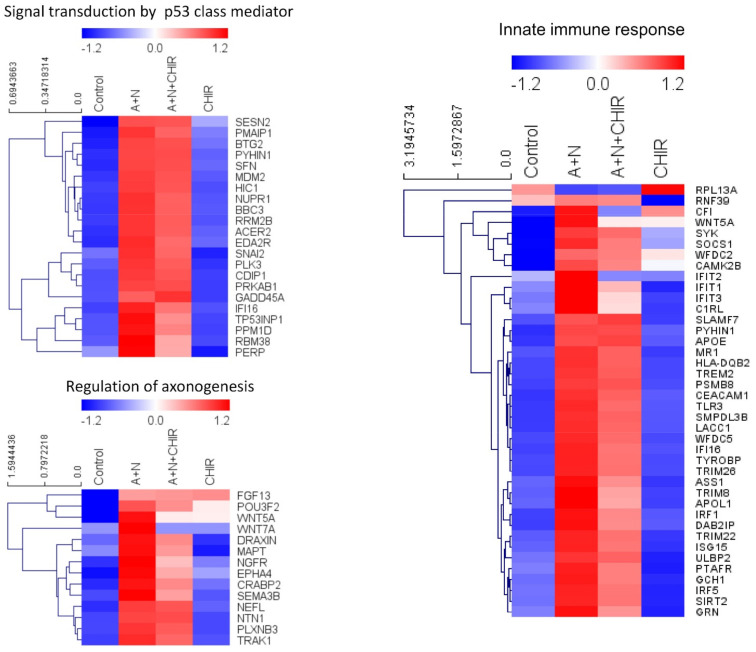
CHIR-98014 attenuates the expression of a subset of genes stimulated by treatment with actinomycin D + nutlin-3a (A + N). Heatmaps demonstrating the RNA-seq expression of genes belonging to selected Gene Ontology groups in control cells and the cells exposed as indicated. In order to generate heatmaps control sample measurements were averaged across three comparisons.

To visualize the abovementioned results, we presented the impact of CHIR on the expression of genes upregulated by A + N using heatmaps. For the presentation, we selected three groups of genes (Figure 2)—the genes belonging to the gene ontology group “signal transduction by p53 class mediator”, the genes belonging to the group “regulation of axonogenesis” and the genes from the group “innate immune response”. Genes belonging to this group were previously shown by us to be significantly stimulated by A + N [4]. These heatmaps demonstrate that upregulation of many genes is weakly or not affected by CHIR (e.g., *BBC3*, *PMAIP1*), upregulation of others is inhibited (*IFIT1*, *IFIT2*, *IFIT3*, *WNT5A*, *WNT7A*, *DRAXIN*, *TREM2*), whereas the expression of small proportion of genes is further upregulated (*GADD45A*). Thus, CHIR does not induce some general inhibition of gene upregulation, but it acts in more targeted fashion, apparently by inhibiting a kinase responsible for upregulation of a subset of genes.

As demonstrated previously [3] exposure of A549 cells to A + N upregulated the expression of proteins belonging to a TREM2 signaling pathway (active in macrophages or microglial cells) starting from TREM2 receptor and encompassing TYROBP, SYK and BLNK proteins. TREM2 receptor has various ligands. One of them is APOE protein, which is an element of lipoprotein particles [8]. Interestingly, transcriptomic analysis demonstrated that the mRNA for APOE was strongly upregulated by A + N (Figure 2). Thus, the exposure of cells to A + N stimulates expression of yet another element of TREM2 signaling pathway-one of its ligands. Before we proceeded to the validation of RNA-Seq results regarding the influence of CHIR on gene expression, we decided to find out whether p53 participates in activation of two genes *APOE* and *BLNK* coding for the elements of TREM2 signaling pathway.

### 2.2. The Activation of APOE Gene Is Attenuated in p53-Deficient Cells

**Figure 3:**
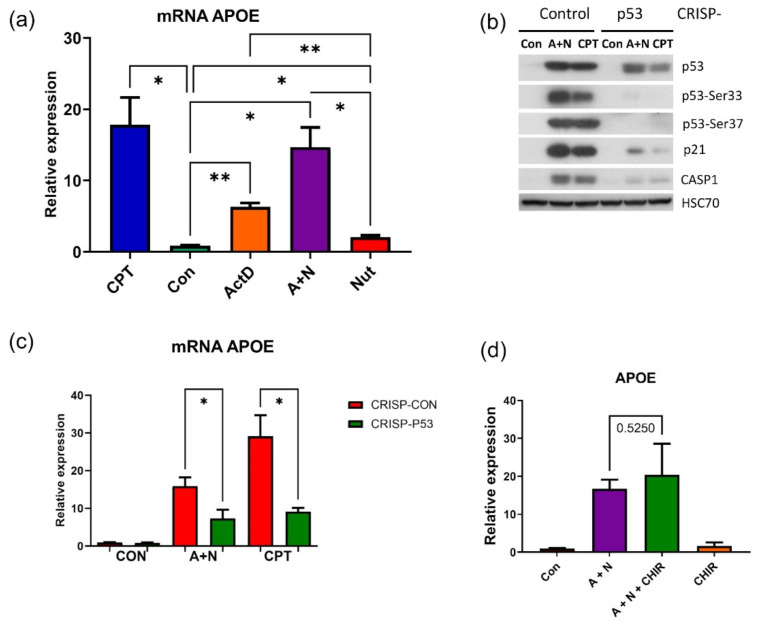
*APOE* is regulated in p53-dependent fashion. (**a**) Measurement of relative *APOE* mRNA level in A549 cells exposed to indicated substances or their combinations for 30 h. CPT-camptothecin, ActD-actinomycin D, Nut-nutlin-3a. The mean and standard deviation from three biological replicates performed in triplicate are presented, *p* values calculated by Brown-Forsythe and Welch’s ANOVA tests (* *p* < 0.05, ** *p* < 0.01) (**b**) Expression of indicated proteins (or p53 phosphorylated on Ser33, Ser37) in p53 knockdown (CRISP) A549 cells or in controls for knockdown. Cells were exposed to A + N or CPT for 30h. (**c**) Relative APOE mRNA levels in p53 knockdown cells (CRISP-p53) or the control cells for knockdown (CRISP-Con) exposed to A + N or CPT for 30 h. The results represent mean and standard deviation from three independent experiments in triplicate, *p* values calculated by Brown-Forsythe and Welch’s ANOVA tests (* *p* < 0.05). (**d**) The level of *APOE* mRNAs measured by semi-quantitative real-time PCR of samples isolated from mock-treated A549 cells (Con) or from cells incubated for 30 h as indicated. The mRNA level in the control cells was defined as 1 (means and standard deviations from three biological replicates). The concentration of CHIR-98014 was 1 µM. Statistical differences in expression were as calculated using unpaired *t* test with Welch’s correction (*p* = 0.5250).

To validate and extend the results of transcriptome sequencing regarding the expression of *APOE* we performed RT-PCR. The RNA samples were isolated from A549 cells treated as demonstrated on Figure 3a. The expression of *APOE* mRNA was stimulated by exposure of cell to strong p53 activators like camptothecin (CPT) or A + N (Figure 3a). The exposure to nutlin-3a, which weakly activates p53, induced hardly any upregulation of *APOE* mRNA.

Next, we tested how the knockdown of p53 influenced the expression of *APOE*. In our previous experiments we employed a model, where the expression of p53 was attenuated by shRNA directed against p53 mRNA. The coding sequence for shRNA was delivered by lentiviral vectors [13]. Because we are studying innate immunity genes here, we wanted to avoid modifying cells using viral vectors. Moreover, it is impossible to select p53-knockout clones from cells with p53 expression inhibited by shRNA. Hence, we employed a different method for making p53-deficient cells. The double-nickase plasmid from Santa Cruz Biotechnology was used to introduce double-strand break in DNA coding for the N-terminal part of p53. The break was repaired in random fashion, what generated mixture of cell clones, with various deletions starting from the N-terminal part of p53. In some clones of cells, the p53 was not expressed (knock-out clones, see below). In most cells, the CRISPR/Cas9-generated mutations resulted in p53 forms, which did not express epitopes recognized by antibodies directed against p53 with phosphorylated serines 33 or 37 (these serines are phosphorylated during p53 activation) (Figure 3b). Thus, most cells either do not express p53 or express mutants with extensive damage to the transcription activating domain localized on the N-terminal part of p53 [14]. In most cells, p53 lost its function because it was unable to upregulate a marker gene (*CDKN1A* coding for p21) for p53 activity. They were also unable to efficiently upregulate another p53 target–caspase-1 [3]. Control cells were transfected with double-nickase control plasmid. Thus, we successfully generated another model of p53-deficient cells. Most subsequent experiments were performed on the mixture of clones to avoid clone-specific effects. It can be argued that lack of activation of a gene in p53-knockout clone is due to off-target effect and not due to lack of p53. On the other hand, if a gene can be activated in p53-knockout clone it clearly indicates that the gene is regulated in p53-independent fashion.

In order to find out how *APOE* is regulated in p53-deficient cells, we exposed them either to A + N or to camptothecin (CPT) for 30 h. Using this model, we demonstrated that expression of *APOE* mRNA was significantly attenuated in p53-deficient cells (Figure 3c). Thus, we conclude that *APOE* gene is activated by A + N and CPT in p53-dependent fashion and potentially, it can participate in autocrine stimulation of the TREM2 receptor. The ChIP-Atlas tool [15] did not reveal any p53 peak within *APOE* gene or 1.5 kbp upstream its transcription start site. This suggests that either *APOE* is indirectly regulated by p53 or that p53 response element is located distantly from the gene. In line with transcriptomic data (Figure 2), we found that CHIR did not attenuate *APOE* expression (Figure 3d). Next, we decided to find out if another element of TREM2 pathway-*BLNK* gene is under the control of p53.

### 2.3. BLNK Gene Is Activated by p53

In previous project we noticed that BLNK protein was upregulated in A549 cells exposed to A + N [3]. The protein was also upregulated in cells exposed to camptothecin, or p53 stimulator, nutlin-3a. The regulation of BLNK protein was significantly attenuated in p53-deficient cells [3]. Based on these observations we hypothesized that p53 directly participates in activation of BLNK gene. However, we did not analyze the functioning of a putative p53 response element within *BLNK* promoter.

**Figure 4:**
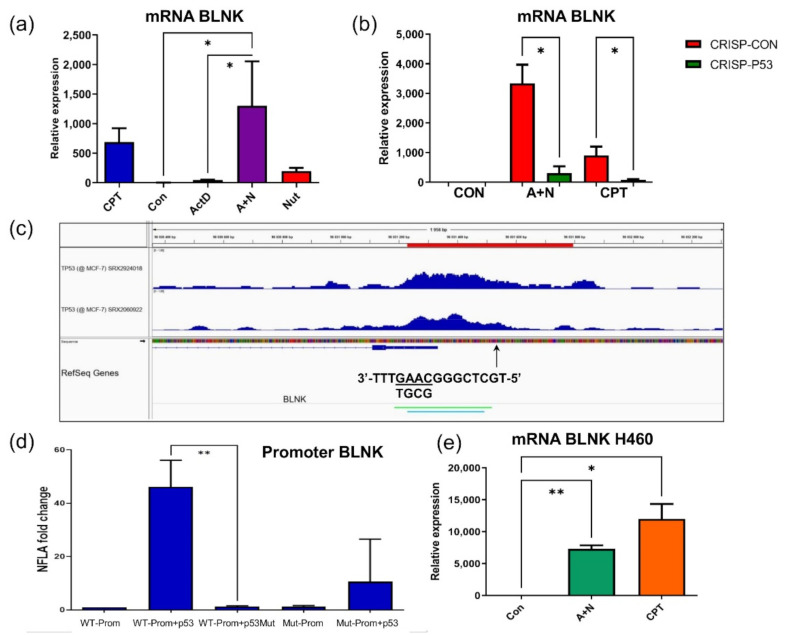
The p53 protein activates expression of *BLNK* gene from response element in promoter region. (**a**) Changes in the levels of *BLNK* mRNA, measured by semi-quantitative real-time PCR of samples isolated from mock-treated A549 cells (Con) or from cells incubated for 30 h as indicated. The mRNA level in the mock-treated population was defined as 1. Results represent means and standard deviations from three biological replicates, *p* values calculated by one-way analysis of variance (ANOVA) with uncorrected Fisher’s LSD (* *p* < 0.05). (**b**) The expression of *BLNK* mRNA determined by semi-quantitative RT-PCR in mock-treated control cells (Con) and in the cells exposed to A + N or CPT for 30 h. The A549 cells with knocked-down p53 are marked as “CRISP-p53”, whereas the controls for knock-down are “CRISP-Con”. The results represent means and standard deviations from three biological replicates, *p* values were calculated by Kruskal-Wallis statistic test with uncorrected Dunn’s test (* *p* < 0.05). (**c**) Genome browser (IGV) views of p53 binding peak in the promoter of *BLNK*. Using ChIP-Atlas tool [15] we imported publically available coverage tracks from two ChIP-Seq experiments aimed at finding p53 binding sites in MCF-7 cell line exposed to ionizing radiation and nutlin (sample ID SRX2924018) or in MCF7 cells treated with nutlin alone (sample ID SRX2060922). The red bar above the tracks marks the promoter sequence cloned in the reporter plasmid. We marked the location of the 3Q p53 response element identified by Tebaldi et al. [16] by an arrow. The 3Q sequence is shown from right to left to match the direction of *BLNK* gene shown by the genome browser. Note that the 3Q element is located near the transcription start site. The position of nucleotide changes generated in mutant promoter are underlined. The nucleotides in the mutation are shown below. (**d**) The fold change of the normalized firefly luciferase activity (NFLA) in U-2 OS cells transfected with the reporter vector coding for firefly luciferase under the transcriptional control of *BLNK* promoter and either empty vector, expression plasmid coding for wild-type p53 or the expression plasmid coding for mutated p53 (Val143Ala). “Mut” prefix indicates the *BLNK* promoter version with the mutated p53 response element as indicated on panel c. The means and standard deviations from three biological replicates performed in triplicate are shown, *p* values calculated by Brown-Forsythe and Dunnett’s T3 multiple comparisons. (**e**) Measurement of relative *BLNK* mRNA level in NCI-H460 cells exposed to indicated substances or their combinations for 30 h, *p* values calculated by Brown-Forsythe and Welch’s ANOVA tests (* *p* < 0.05, ** *p* < 0.01).

The RT-PCR demonstrated that mRNA of BLNK was upregulated more than 1000-fold in A549 cells exposed to A + N (Figure 4a). This is very strong increase, probably due to very low expression of BLNK in untreated cells, what is consistent with the fact that in unstressed cells BLNK expression is limited to some cells of the immune system (primarily B cells). Its mRNA was also upregulated by camptothecin (more than 400-fold) or by nutlin-3a (200-fold). The degree of synergy between actinomycin D and nutlin-3a in stimulation of *BLNK* was very strong considering that actinomycin D acting alone upregulates BLNK mRNA only about 50-fold (Figure 4a). Thus, *BLNK* belongs with *CASP1*, *STING1*, *DRAXIN* and *H19* to the group of genes synergistically upregulated by actinomycin D and nutlin-3a [3,4,5]. Stimulation of *BLNK* expression at mRNA level triggered either by A + N or camptothecin was significantly attenuated in p53-deficient cells (Figure 4b), what is consistent with the aforementioned observation that expression of BLNK protein was strongly attenuated in our earlier model of p53-deficient cells [3]. Strong upregulation of *BLNK* mRNA by treatment with A + N or camptothecin was also observed in NCI-H460 lung cancer cell line, what indicates that activation of the gene by stress is not a peculiarity of A549 cells. Hence, apparently, p53 participates in upregulation of *BLNK* gene. To pinpoint the localization of p53 response element (RE) within *BLNK*, we employed two approaches. First, we searched in the database published by Tebaldi et al. [16] for the presence of p53 RE in *BLNK*. One such sequence (5′-CTATGTGCTCGGGCAAGTTT-3′), was identified within promoter region (Figure 4c). It belongs to the category of three-quarter (3Q) sites, because the first 5 nucleotides of the sequence (underlined) do not match the consensus p53 RE consisting of 20 nucleotide sequence, RRRCWWGYYYRRRCWWGYYY (R-purine, Y-pyrimidine, W-A or T) [16]. The other approach was to look for p53 ChIP-Seq peaks using ChIP-Atlas platform [15]. Using the latter approach, we noticed p53 ChIP-Seq peak located in *BLNK* promoter in MCF7 breast cancer cells either exposed to nutlin-3a or to nutlin-3a and ionizing radiation (Figure 4c). Thus, in some stress conditions, p53 binds to the promoter region of *BLNK*. Interestingly, p53-ChIP-Seq peaks encompass the 3Q p53 RE identified by Tebaldi et al. [16]. We overlapped this 3Q site on the peaks generated by IGV genome viewer (Figure 4c). To further characterize the p53 RE in *BLNK* promoter, we cloned DNA sequence containing 3Q site and ChIP-Seq peaks in pGL3-Basic reporter vector coding for firefly luciferase activity. In mutant version of the promoter the consensus site was mutated as indicated on Figure 4c. The wild-type version of the plasmid was transfected to U-2 OS cells either with empty expression vector, with plasmid producing wild-type p53 or with plasmid producing mutant p53 (Val143Ala), which lost ability to bind DNA and transactivate p53-regulated genes. Wild-type p53 stimulated activity of *BLNK* promoter 45-fold. Mutant p53 was not able to do so. Moreover, promoter with mutant version of p53 response element weakly responded to expression of wild-type p53. In summary, the data published by Tebaldi et al. [16], the presence of ChIP-Seq peaks and the experiments performed by us indicate that *BLNK* is p53-regulated gene controlled from at least one RE present in *BLNK* promoter.

### 2.4. CHIR-98014 Attenuates Activation of p53, but Sensitizes Cells to Apoptosis

**Figure 5:**
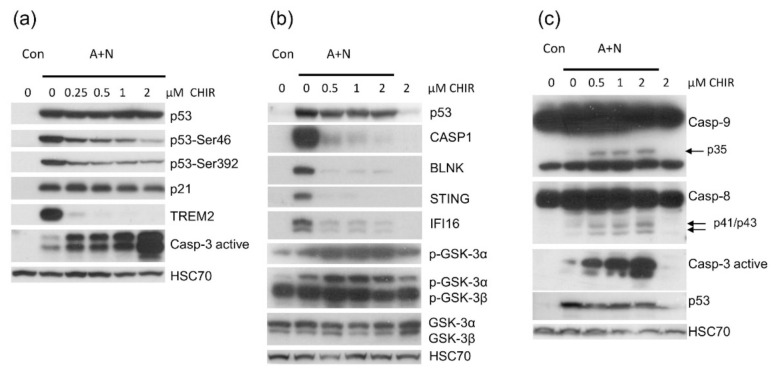
CHIR-98014 promotes activation of executioner caspase of apoptosis in A549 cells exposed to A + N. (**a**) Western blot showing expression of indicated proteins (or p53 phosphorylated on Sr46 or Ser392) in A549 cells mock-treated (Con) or incubated for 48 h with actinomycin D and nutlin-3a (A + N) with or without addition of CHIR-98014 at indicated concentrations. The level of active caspase-3 is also indicated. (**b**) Expression of proteins in A549 cells exposed as indicated. The phosphorylation of GSK-3α on Ser21 was assessed using antibody anti-phospho GSK-3α (Ser21). The phosphorylation of GSK-3β on Ser9 was assessed using antibody anti-phospho GSK-3β (Ser9), which also detects phosphorylated GSK-3α (upper band). (**c**) The expression of cleaved forms of caspase-8 and caspase-9 (arrows) and the activated caspase-3 in A549 cells exposed as indicated. In all experiments from this figure cells were incubated with the substances for 48 h.

Considering that p53 is major inducer of pro-apoptotic genes and that our transcriptomic analysis demonstrated that CHIR attenuates the activation of some p53 target genes, we tested how CHIR modulates apoptosis in cells exposed to A + N. We performed the experiment exposing A549 cells to A + N and to A + N with increasing concentrations of CHIR. Expectedly, we found that CHIR attenuated phosphorylation of p53 on Ser46 and Ser392 (markers of p53 activation) (Figure 5a). Consistent with previous findings, upregulation of TREM2 was prevented even by the 0.25 µM concentration of CHIR, whereas the activation of gene (CDKN1A) coding for p21 protein, which mediates the cell cycle inhibition triggered by p53, was not influenced. CHIR sensitized cells to apoptosis (measured by the level of active caspase-3) induced by A + N in spite of attenuated activation of p53. The effect was visible even at sub-micromolar concentrations of CHIR and was prominent when the concentration of the inhibitor was increased to 2 μM (Figure 5a).

Two p53-regulated genes responded in different fashion to the inhibitory effect of CHIR (Figure 5a). Upregulation of TREM2 was blocked, whereas the upregulation of p21 protein was unchanged. Thus, CHIR impacts on p53 target genes in different fashion, what is consistent with our transcriptomic results. To find out how other p53-regulated genes respond to CHIR we exposed cells to A + N and CHIR and we examined protein expression by Western blotting (Figure 5b). We selected CASP1 because its expression is enormously activated by A + N [4], BLNK and STING (TMEM173) because we wanted to better characterize their expression as newly detected, p53-regulated genes. IFI16 is innate immunity gene and well-known p53 target presented on the heatmap (Figure 2). The activation of BLNK, CASP1, STING and IFI16 was prevented by CHIR in sub-micromolar concentration (Figure 5b). Thus, CHIR does not generally block the activation of p53 but it acts more specifically towards a subset of p53-regulated genes.

Biochemical analyses show that CHIR can inhibit both isoforms of GSK-3 kinase α and β [9]. The activities of these kinases are regulated by phosphorylation. There is inhibitory phosphorylation on residue Ser21 for GSK-3α and on residue Ser9 for GSK-3β. We employed two antibodies to test the phosphorylation status of these amino acids. One antibody was directed against Ser9 GSK-3β but may cross-react with phospho-GSK-3α, whereas the other antibody was directed against phospho-GSK-3α (Ser21). We also employed antibody, which recognized the total amount of both isoforms. Western blotting gave consistent results demonstrating that in our experimental conditions CHIR induces inhibitory phosphorylation of GSK-3α at Ser21 (Figure 5b).

To further explore the mechanism of apoptosis-promoting activity of CHIR, specifically to find out, which pathway of apoptosis was activated, we monitored the cleavage of caspase-8 (participating in receptor-induced apoptosis) and caspase-9 (participating in mitochondria-induced apoptotic pathway). We observed that both caspases were cleaved in cells exposed to A + N with CHIR what is consistent with their activation. Thus, both apoptosis-inducing pathways were stimulated in cells exposed to A + N in presence of CHIR (Figure 5c).

**Figure 6:**
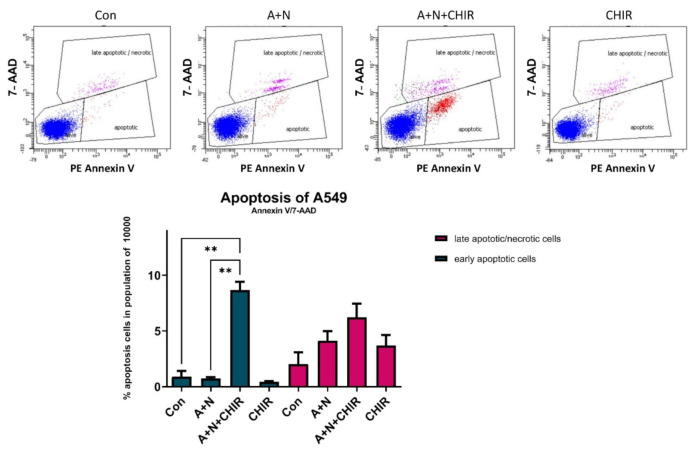
Flow cytometry analysis confirms ability of CHIR-98014 to induce apoptosis in cells exposed to A + N. Cytometric analysis of cell populations mock-treated (Con) or exposed to A + N, A + N with CHIR-98014 or CHIR-98014 alone for 48 h. Viable cells are 7-AAD (7-Amino-Actinomycin) negative and PE Annexin V negative; cells in early apoptosis are PE Annexin V positive and 7-AAD negative, whereas late apoptotic or dead cells are both PE Annexin V and 7-AAD positive. The graph below shows the frequency of indicated cell types calculated from three biological repeats (*p* values calculated by Brown-Forsythe and Welch’s ANOVA tests (** *p* < 0.01).

To measure the frequency of apoptotic cells generated in response to our treatment modalities, we performed cytometric analysis (Figure 6). Its results are consistent with Western blotting. In the cell population exposed to A + N we did not observe increased frequency of early apoptotic cells, we noticed however higher frequency of cells in late apoptosis or necrosis, although the increase was not statistically significant. The significant increase of frequency of early apoptotic as well as late apoptotic/necrotic cells was observed in the population of cells exposed to A + N with CHIR (Figure 6). Thus, three different methods (Western blotting, cytometry, RNA-Seq-Figure 1d) clearly indicated that addition of CHIR to A549 cells exposed to A + N promotes apoptosis.

### 2.5. CHIR-98014 Strongly Attenuates Activation of Antiapoptotic Genes Stimulated by p53, but Does Not Markedly Change the Activation of Known p53-Regulated Pro-Apoptotic Genes

For a particular cell, the apoptosis is an all-or-nothing phenomenon. Entering into apoptosis is governed by the balance of pro- and anti-apoptotic signals present in a given cell. The signals consist of the levels of pro- and anti-apoptotic proteins as well as their pro- and anti-apoptotic posttranslational modifications. Thus, the presence of CHIR may tip the balance toward apoptosis, e.g., by promoting expression of pro-apoptotic genes and/or by repression of anti-apoptotic genes. The p53 protein is best known from the stimulation of pro-apoptotic genes, but there are reports showing that it can activate some anti-apoptotic genes, e.g., we observed [5], that A + N stimulated, in p53-dependent fashion, the expression of H19 gene producing long non-coding RNA molecules with strong anti-apoptotic activity [17].

**Figure 7:**
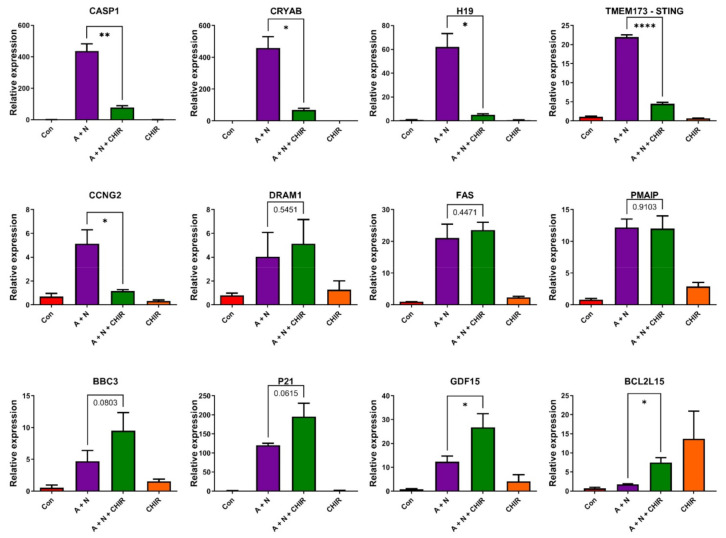
Genes response to the treatment with CHIR-98014 is not uniform. Changes in the levels of mRNAs of indicated genes, measured by semi-quantitative real-time PCR of samples isolated from mock-treated A549 cells (Con) or from cells incubated for 30 h as indicated. The mRNA level in the mock-treated population was defined as 1. The concentration of CHIR-98014 was 1 µM. Results represent means and standard deviations from three biological replicates. Statistical differences in expression were calculated using unpaired *t* test with Welch’s correction (* *p* < 0.05, ** *p* < 0.01, **** *p* < 0.0001).

The other anti-apoptotic, p53-regulated gene, which we are aware of, CRYAB codes for a chaperone protein known as crystallin alpha B [18]. Its anti-apoptotic activity was detected in various model systems [19,20]. Hence, we decided to study by RT-PCR how CHIR influences the expression of p53-regulated genes involved in innate immunity (*CASP1*, *TMEM173*), genes coding for anti-apoptotic molecules (CRYAB, H19), genes inhibiting the cell cycle progression (*CDKN1A*, *CCNG2*), gene coding for autophagy-promoting protein (*DRAM1*) and the genes coding for major pro-apoptotic proteins (*BBC3*, *FAS*, *PMAIP1*). The cells were treated with A + N, with A + N and additionally with CHIR and with CHIR alone (Figure 7).

In agreement with Western blotting data, we found that CHIR prevented strong upregulation of *CASP1* and *STING1* (*TMEM173*). The inhibitor also prevented upregulation of anti-apoptotic *H19* and *CRYAB* genes and one of the genes regulating the cell cycle (*CCNG2*). Interestingly, CHIR did not significantly influence the expression of cell cycle regulator *CDKN1A* coding for p21 protein (what is also consistent with Western blot data, Figure 5a), and did not influence or even increase the expression of pro-apoptotic *BBC3*, *FAS* and *PMAIP1*. We compared our RT-PCR data with the transcriptomic data and we noticed that for all genes presented on Figure 7 both techniques gave consistent results indicating that our transcriptomic analysis used as a screening method for differentially expressed genes gave reliable results.

We also studied the expression of gene, *GDF15*, which is regulated by p53 and which positively regulates apoptosis [21]. Our transcriptomic data demonstrated that CHIR intensified the effect of A + N treatment on *GDF15* gene expression. The RT-PCR analysis confirmed these results. While A + N alone upregulated *GDF15* expression about 12-fold, addition of CHIR doubled this effect (Figure 7). Stimulating the expression of *GDF15* in presence of A + N may be another factor by which CHIR promotes apoptosis. And finally, we found a pro-apoptotic gene from BCL family [22], *BCL2L15* (selected from transcriptomic data), whose expression was barely stimulated by A + N but was significantly increased by CHIR either acting in presence of A + N or when applied alone (Figure 7). Thus, these results are consistent with our hypothesis, that CHIR promotes the apoptosis by tipping the balance of pro- and anti-apoptotic signals in favor of pro-apoptotic ones.

### 2.6. CHIR Sensitizes Other Cell Lines to Apoptosis

**Figure 8:**
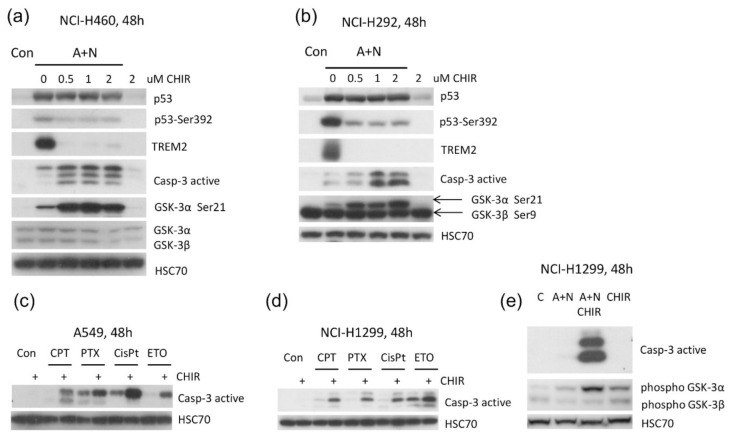
CHIR-98014 promotes activation of caspase-3 and represses TREM2 in cell lines other than A549. (**a**,**b**) Two lung cancer cell lines NCI-H460 and NCI-H292 with wild-type p53 were treated as indicated. The expression of selected proteins or their forms phosphorylated on indicated amino acids was examined by Western blotting. (**c**,**d**) The expression of active caspase-3 in cells exposed to anticancer drugs acting alone or in combination with CHIR-98014. The following concentrations of drugs were used: camptothecin (CPT) 1 µM, paclitaxel (PTX)-5 µM, cisplatin (CisPt)–20 µM, etoposide (ETO)–20 µM, CHIR-98014 -1 µM. (**e**) The expression of active caspase-3 and phosphorylated forms of GSK-3α or GSK-3β in p53-null NCI-H1299 cells exposed as indicated. The concentration of CHIR-98014 was 2 µM.

To find out if the apoptosis-promoting activity of CHIR is not limited to A549 cell line, we repeated the treatments on other cells with wild-type p53. We used cells previously studied by us in the context of p53 activation [2], NCI-H292 and NCI-H460 derived from lung cancers. We performed the treatment with A + N and CHIR as demonstrated on Figure 8a,b. We noticed that in both cell lines: (i) A + N treatment strongly upregulated expression of TREM2, (ii) upregulation of TREM2 was blocked by sub-micromolar concentration of CHIR, (iii) treatment with CHIR sensitized cells exposed to A + N to apoptosis measured as activation of caspase-3, (iv) the treatment with A + N and with CHIR strongly induced inactivating phosphorylation of GSK-3α (on Ser21). Thus, attenuated activation of some p53-regulated genes, e.g., TREM2, stimulation of apoptosis and inactivating phosphorylation of GSK-3α take place in various cell lines in response to treatment with CHIR in the presence of A + N. The underlying molecular mechanism probably operates in many cell types.

We also tested if CHIR can enhance apoptosis triggered by widely-used chemotherapeutic agents. To this end, we exposed A549 cells to camptothecin, paclitaxel, cisplatin and etoposide acting alone or in combination with CHIR (Figure 8c). CHIR enhanced pro-apoptotic activity of all drugs, however, this effect was more prominent in case of camptothecin and etoposide. In principle, CHIR can induce apoptosis by modulating activity of p53 or through changing the expression of other modulators of apoptosis like BCL2L15. To find out whether the sensitization to apoptosis depends on the activity of p53, we performed our experiment on p53-null cell line NCI-H1299. We noticed very strong induction of caspase-3 activity in cells exposed to CHIR in the presence of A + N (Figure 8e). Clearly, this inhibitor can sensitize cells to apoptosis in the absence of p53. Interestingly, also in this cell line, CHIR augmented apoptosis induced by various chemotherapeutic agents (Figure 8d).

### 2.7. Knock-down of Kinases Inhibited by CHIR-98014–GSK3A or GSK3B Does Not Mimic the Activity of CHIR

It is not uncommon for a kinase inhibitor to have an off-target effect. Hence, we decided to use CRISPR/Cas9 technology to observe how the knock-down of *GSK3A* or *GSK3B* expression would impact on the expression of TREM2 and active caspase-3 in cells exposed to A + N. We used this experimental endpoint because inhibition of TREM2 upregulation was one of the strongest effects of CHIR-98014 (Figure 5a and Figure 8a,b).

**Figure 9:**
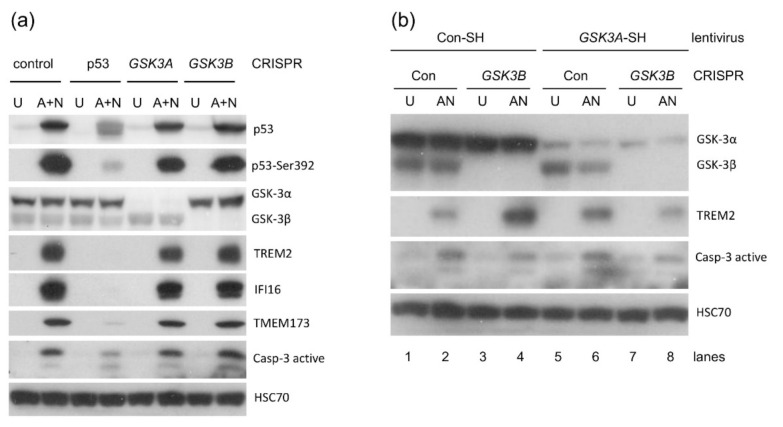
Downregulation of GSK-3 does not mimic the effect of CHIR-98014 on TREM2 expression or caspase-3 activation. (**a**) The expression of selected proteins in cells with the expression of either GSK-3α, GSK-3β or p53 downregulated by CRISPR/Cas9 technology. Control cells for knockdown were also used. The cells were either mock-treated (U) or exposed to A + N for 48 h. (**b**) Expression of TREM2 and active caspase-3 in cells with double knockdown of *GSK3A* and *GSK3B*. The cells with *GSK3B* expression knocked-down by CRISPR/Cas9 technology or the controls for knockdown were transduced either with lentivirus producing the shRNAs directed against *GSK3A* mRNA or with the control lentivirus. We obtained four cell lines as demonstrated. One of the cell lines had strong downregulation of both *GSK3A* and *GSK3B*. The cells were either mock-treated (U) or exposed to A + N for 48 h.

To knock down either *GSK3A* or *GSK3B*, we used commercially available system of plasmids expressing Cas9 nuclease and a pair of guide RNAs directed to a target gene. Using two gRNA sequences increases specificity of gene disruption. In order to estimate the degree of gene silencing we performed Western blot using the antibody, which can detect both GSK-3α (upper band) and GSK-3β (lower band, Figure 9a). We generated populations with majority of cells not expressing either GSK-3α or GSK-3β. We did not isolate pure clones in order to avoid clone-specific effects, which frequently occur during cloning of mammalian cells in culture. Similar procedure was used to generate the knockdown of *TP53* gene as mentioned previously (Figure 3b). Unexpectedly, we found that in cells with significantly reduced expression of either GSK-3α or GSK-3β, the upregulation of TREM2 induced by A + N or activation level of caspase-3 was very similar to control cells (Figure 9a). Thus, the suppression of one isoform of GSK-3 influenced neither the expression of TREM2 nor activation of caspase-3. In this scenario, the remaining kinase can substitute the mutated one in mediating the molecular changes leading to TREM2 upregulation and caspase-3 activation. Alternatively, in our experiments, CHIR targeted molecule other than GSK-3α or GSK-3β.

To find out if one isoform of GSK-3 can substitute the other in activation of TREM2 or activation of caspase-3 we engineered cell line with significant knockdown of both isoforms. We selected cells with GSK-3β expression knocked-down by CRISPR/Cas9 method (Figure 9a) and we transduced it with lentiviruses expressing shRNA molecules against mRNA of GSK-3α. We included proper controls (GSK-3β knockdowns transduced with control lentivirus, and control knockdown cells transduced with GSK-3α lentivirus). In experimental cells we had very strong knockdown of both isoforms. In spite of this, in these cells the upregulation of TREM2 by A + N was as strong as in control cells expressing both kinases (Figure 9b). We conclude, that CHIR prevents the upregulation of TREM2 by a mechanism other than inhibiting of GSK-3. This gives us a case for cautious interpretation of the results of our experiments concerning the role of GSK-3 in modulation of gene expression and induction of apoptosis. Kinase inhibitors frequently have some off-target effects. If not GSK-3, then what is the target of CHIR, which regulates apoptosis and promotes the expression of some p53-target genes? At this stage we do not have answer to this question. It is plausible that this kinase modifies p53 in a way, which helps stronger binding to selected gene regulatory elements. Alternatively, p53 modified by this kinase better interacts with co-activators playing crucial roles in stimulation of selected, p53-regulated genes. We selected another compound named PHA793887, which inhibits cyclin-dependent kinases CDK2, CDK5 and CDK7. All three kinases are able to phosphorylate p53 [23,24,25]. In sub-micromolar concentration (0.5 μM) PHA793887 inhibited upregulation of TREM2, BLNK, STING1 and CASP1 (Figure S1). The upregulation of p21 protein was attenuated but not prevented. This inhibitor, in the presence of A + N, also induced the activation of caspase-3. Thus, considering the effects on p53-regulated genes and on activity of caspase-3 both compounds CHIR-98014 and PHA-793887 demonstrate similar activities probably due to inhibition of the same kinase.

### 2.8. CHIR Prevents Upregulation of Both p53-Regulated and p53-Independent Genes in Cells Exposed to A + N

Our previous experiments and current transcriptomic data show that A + N treatment induces expression of many innate immunity genes. Expression of some of them is regulated by p53 (*TMEM173*, *NLRP1*, *NLRX1*), whereas the role of p53 in activation of others is ambiguous, e.g., *IFIT1*, *IFIT3* [4]. IFIT1 and IFIT3 genes can be regulated by IRF1 transcription factor [26], which is upregulated by interferon γ but also by DNA damaging agents independently of p53 [27]. Because our transcriptomic results demonstrated that IRF1 is upregulated by A + N (Figure 2), we hypothesized that in response to A + N, IRF1 is stimulated independently of p53 and that IRF1 subsequently stimulates IFIT1, IFIT3 and probably other innate immunity genes.

**Figure 10:**
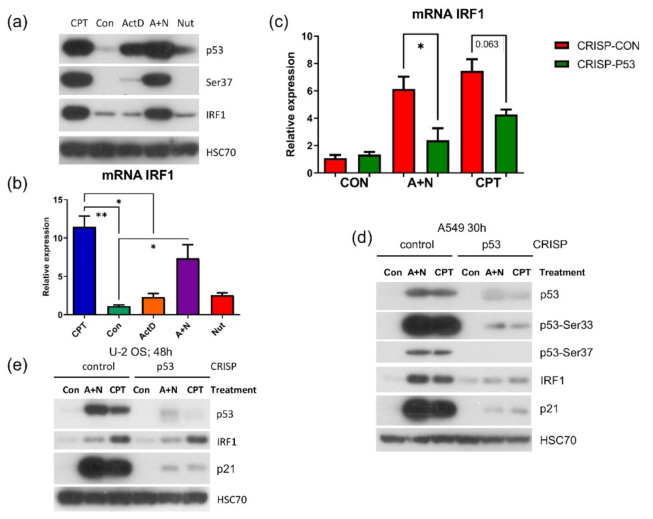
In A549 cells p53 participates in the induction of IRF1 transcription factor. (**a**) Western blot demonstrating expression of p53, its form phosphorylated on Ser37 and of IRF1 protein in A549 cells treated as indicated for 48 h. (**b**) Measurement of relative IRF1 mRNA level in A549 cells exposed to indicated substances or their combinations for 30 h. Statistical differences in expression was calculated by Kruskal-Wallis statistic test with uncorrected Dunn’s test (* *p* < 0.05) (**c**) Relative IRF1 mRNA levels in p53 knockdown cells (CRISP-p53) or the control cells for knockdown (CRISP-Con) exposed to A + N or CPT for 30 h (*p* values were calculated by Kruskal-Wallis statistic test with uncorrected Dunn’s test (* *p* < 0.05, ** *p* < 0.01). (**d**) Expression of indicated proteins (or p53 phosphorylated on Ser33 or Ser37) in p53 knockdown (CRISP) A549 cells or in controls for knockdown. Cells were exposed to A + N or CPT for 30h. (**e**) Expression of p53, IRF1 and p21 in U-2 OS cells with p53 expression knocked-down by CRISPR/Cas9 method employed to knock down p53 in A549 cells.

Thus, we validated the results of transcriptomic analysis concerning IRF1 using Western blotting (Figure 10a) and RT-PCR (Figure 10b). Expression of this gene can be stimulated concomitantly with strong p53 activation. Surprisingly, upregulation of IRF1 at mRNA and protein level was attenuated in p53-deficient cells (Figure 10c,d). Thus, in A549 cells exposed to A + N, p53 participates in stimulation of IRF1, which contrasts with the results of Tanaka et al. [27] demonstrating that in response to DNA damage, IRF1 is activated independently of p53. We repeated this experiment on engineered p53-deficient U-2 OS cell line (Figure 10e). Surprisingly, in these cells, IRF1 was not stimulated by A + N and its activation by camptothecin was not influenced by p53 status. Thus, definitely, IRF1 in some cells (e.g., U-2 OS) can be stimulated by DNA damaging agents (e.g., camptothecin) independently of p53, what is consistent with the conclusions of Tanaka et al. [27], whereas in other cell types (e.g., A549) and in some stress conditions (A + N), p53 is indispensable for IRF1 activation.

**Figure 11:**
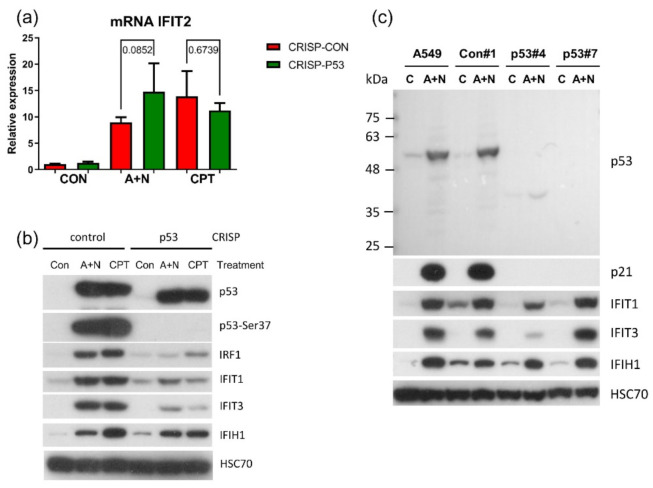
A subset of innate immunity genes can be activated by A + N without the assistance of p53. (**a**) Measurement of relative IFIT2 mRNA level in p53 knockdown cells (CRISP-p53) or the control cells for knockdown (CRISP-Con) exposed to A + N or camptothecin (CPT) for 30 h. Statistical differences in expression were calculated by Kruskal-Wallis statistic test with uncorrected Dunn’s test. (**b**) Expression of indicated proteins (or p53 phosphorylated on Ser37) in p53 knockdown (CRISP) A549 cells or in controls for knockdown. Cells were exposed to A + N or CPT for 48 h. (**c**) The expression of indicated proteins in clones (#4 and #7) of p53 knockout cells from A549 cell line. The control clone for knockout (#1) as well as the parental A549 cell line were used as controls. The cells were mock-treated (C) or exposed to A + N for 48 h. We presented enlarged image of p53 blot to demonstrate that no protein versions with deletion or insertion are expressed.

IRF1 can stimulate expression of IFIT1 and IFIT3 [26]. Our previous experiments with earlier model of p53 knockdown by lentivirus-delivered shRNAs, did not convincingly demonstrate that p53 was involved in activation of IFIT1 and IFIT3 [4]. We decided to employ our new CRISPR/Cas9 model of p53 knockdown to determine the role of p53 in expression IFIT1 and IFIT3. We also tested two other innate immunity genes, whose expression was stimulated by A + N in transcriptomic analysis-IFIH1 and IFIT2. The new model demonstrated that p53 was involved in upregulation of IFIT1 and IFIT3 (Figure 11b), but did not participate in stimulation of IFIT2 and IFIH1 (Figure 11a,b).

To further analyze the role of p53 in activation of innate immunity genes we selected two clones of A549 cells, which do not express p53 protein (the knockout clones). We also examined one control clone for knockout. These clones were exposed to A + N together with the unmodified A549 cells (Figure 11c). Surprisingly, in both p53 knockout clones exposure to A + N resulted in upregulation of IFIT1 and IFIT3. Thus, apparently, in response to A + N these two genes can be activated by two mechanisms, one that relies on p53 and the other, which is p53-independent.

**Figure 12:**
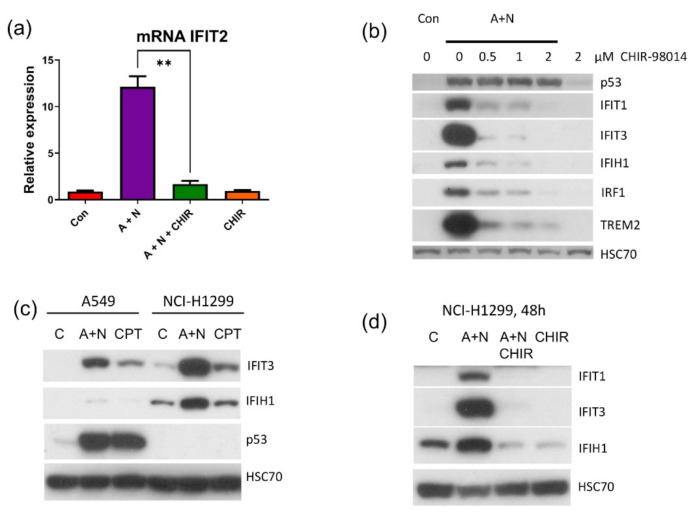
CHIR-98014 attenuates the expression of innate immunity genes stimulated by A + N without the assistance of p53. (**a**) Relative expression of IFIT2 mRNA in cells exposed as demonstrated for 30 h (CHIR, 1µM). Statistical differences in expression were calculated using unpaired *t* test with Welch’s correction (** *p* < 0.01). (**b**) The expression of indicated proteins in A549 cells exposed to A + N or A + N with various concentrations of CHIR-98014 for 48 h. (**c**) The expression of p53, IFIT3 and IFIH1 proteins in cells with wild-type p53 or expressing no p53. The cells were exposed to A + N or camptothecin (CPT) for 48 h. (**d**) The expression of the innate immunity proteins in p53-null NCI-H1299 cell line exposed as indicated (CHIR, 2 µM).

To validate the results of transcriptomic analysis presented on Figure 2 concerning the role of CHIR in regulation of selected innate immunity genes, we performed RT-PCR for IFIT2 and Western blotting for IFIT1, IFIT3, IFIH1 and IRF1 (Figure 12a,b). Based on these results, we conclude that CHIR strongly attenuates the upregulation of these genes induced by A + N. Because IFIT1, IFIT3 and IFIH1 can be stimulated independently of p53 in A549 cells, we wanted to use another model, to demonstrate that these genes can be activated without the assistance of p53. We employed p53-null cell line NCI-H1299. We exposed NCH-H1299 cells to A + N or camptothecin and we examined expression of IFIT3 and IFIH1 (Figure 12c). These proteins accumulated following treatment with A + N (but not camptothecin). Moreover, the upregulation of IFIT1, IFIT3 and IFIH1 was attenuated by CHIR in these p53-null cells (Figure 12d). Thus, this inhibitor can block not only a subset of p53-regulated genes but also genes activated without assistance of p53 in cells exposed to A + N.

## 3. Discussion

We screened by transcriptome sequencing for genes upregulated by p53 strongly activated with the combination of actinomycin D and nutlin-3a (A + N). Moreover, we screened for genes whose upregulation by A + N was modulated by CHIR, a compound considered as specific inhibitor of both isoforms (α and β) of GSK-3 kinase [9]. The working hypothesis was that GSK-3 phosphorylates p53 and modifies its activity in a way that allows for strong activation of some p53-regulated genes like TREM2. This hypothesis was based on our earlier observations [3]. We wanted to find out what other genes are regulated by GSK-3 through modification of p53. We validated the transcriptomic results for selected genes using RT-PCR or Western blotting. The result of sequencing itself and the outcome of its bioinformatic analysis were sound, judging by the fact that the overrepresentation analysis noticed activation of p53 signaling pathway (Figure 1). The list of genes upregulated by A + N can be used as a collection of candidate p53-regulated genes for more in-depth analysis. For example, we selected *BLNK* gene and we demonstrated that p53 participates in its upregulation. Moreover, we found p53 response element located in *BLNK* promoter (Figure 4). BLNK protein may be a part of the signaling pathway starting from TREM2 protein. Upon ligand binding, TREM2 indirectly activates SYK kinase. This kinase, in turn, utilizes BLNK protein for selection of downstream targets [28]. The appearance of BLNK protein in A549 lung cancer cells exposed to A + N was very surprising, because the expression of *BLNK* gene is limited to B cells, plasmacytoid dendritic cells and microglial cells. Most of our knowledge about functioning of BLNK is derived from studies on B-cells. The protein is an adapter linking activation of B-cell receptor and SYK kinase to the activation of phospholipase Cγ2 and Ca^2+^ influx to cytosol [29]. Our observations indicate that, for some reason, function of BLNK protein is required in lung cancer cells in stress conditions elicited by treatment with A + N.

Interestingly, activation of *BLNK* was visible in transcriptomic data although it did not reach statistical significance. This illustrates that for the screening purposes, or picking out candidate p53-regulated genes for extended analyses, our transcriptomic results can be used without strict adherence to the set level of statistical significance. Similarly, we demonstrated, that p53 participates in upregulation of *APOE* gene (Figure 3). Curiously, it appears that p53 controls at least two genes, *TREM2* and *APOE*, whose polymorphisms are strongly associated with the risk of Alzheimer’s disease [3,8,30].

Because CHIR attenuated activation of some p53-regulated genes and inhibited p53 phosphorylation, initially we hypothesized that this inhibitor would attenuate apoptosis of cells exposed to A + N. To our surprise, we found elevated level of activated caspase-3 (Figure 5a). Thus, in spite of attenuated p53 activation, the cells were more frequently undergoing apoptosis what was also demonstrated by flow cytometry analysis (Figure 6). CHIR acting alone did not induce apoptosis (Figure 5c and Figure 6). Because we detected activating proteolytic cleavage of both, caspase-8 and caspase-9, we conclude that both apoptosis-inducing pathways, intrinsic and death receptor-driven, are activated in cells exposed to A + N and CHIR (Figure 5c).

The transcriptomic data gave us unique opportunity for creating hypotheses concerning the apoptosis-promoting activity of CHIR. We noticed that CHIR prevented strong upregulation of two genes, known to be p53-targets, which have strong anti-apoptotic activity-*H19* [5,31] and *CRYAB* [18,32] (Figure 7). Moreover, CHIR enhanced upregulation of *GDF15*-another p53 target with pro-apoptotic activity [33,34] and CHIR (but not A + N) strongly stimulated the expression of *BCL2L15*, which codes for pro-apoptotic protein belonging to Bcl-2 family [22]. It is not known, which genes have critical role in the induction of apoptosis triggered by A + N with CHIR. These are probably not p53-regulated genes because CHIR shows similar apoptosis-promoting activity in p53-null cell line NCI-H1299 (Figure 8d,e). Moreover, CHIR enhances pro-apoptotic activity of other chemotherapeutic agents, which are routinely used in clinics like cisplatin, etoposide, paclitaxel or camptothecin (Figure 8c,d). The pro-apoptotic activity of CHIR in mono-treatment was observed by others [35]. It was associated with mitochondrial dysfunction and ROS generation after relatively long treatment (72 h). Moreover, CHIR together with an experimental substance inhibiting BET family proteins showed remarkable therapeutic effects in vitro and in vivo acting synergistically against liver cancer cells [36].

Considering that CHIR is regarded as specific inhibitor of GSK-3 kinases [9] our results suggested that one of its isoforms (or both) are involved in regulation of apoptosis and gene expression in cells exposed to A + N. One observation suggested that GSK-3α may be involved, because exposure to CHIR was associated with appearance of inhibitory phosphorylation of this enzyme on Ser21 (Figure 5b and Figure 8a,b). In order to test whether the impact of CHIR is through inhibition of GSK-3, we generated cell lines with the GSK-3 expression significantly attenuated. We silenced either *GSK3A* or *GSK3B* genes, but to our surprise the knockdown had no visible influence on the expression of the gene strongly inhibited by CHIR-*TREM2*. The knockdown did not sensitize to apoptosis either (Figure 9a). Thus, the knockdown of a single isoform of GSK3 did not mimic the activity of CHIR. It was plausible that the kinases could substitute each other in relevant activity. However, the knockdown of both isoforms influenced neither *TREM2* expression nor caspase-3 activation. Thus, we cannot argue that the effects of CHIR on apoptosis or gene expression that we observed in this work are mediated through the inhibition of GSK-3. It is safer to say that the effects are mediated through the inhibition of a kinase or even several kinases targeted by CHIR.

Our previously published data and current transcriptomic analysis demonstrated that A + N activated expression of several dozen innate immunity genes (Figure 2 and [4]). One of the genes selected through transcriptomic analysis is *IRF1*. It codes for a transcription factor controlling the expression of other innate immunity genes [37]. Thus, by upregulating IRF1, the A + N combination could control the expression of many other genes. An intriguing possibility was that through activation of IRF1, the treatment with A + N could act independently of p53, because IRF1 gene was found to be stimulated by DNA damaging agents without the assistance of p53 [27]. To our surprise, we observed that in p53–deficient A549 cells, IRF1 protein accumulated neither following exposure to A + N nor camptothecin. Upregulation of its mRNA was also attenuated (Figure 10). Thus, definitely p53 participates in stimulation of IRF1 in A549 cells. However, p53 cannot activate this gene in U-2 OS cell line. In A549 cells IRF1 is upregulated only when p53 is strongly activated through phosphorylation (Figure 10a). In U-2 OS cells there is a deficiency in p53 activation in some experimental conditions [2]. We speculate that this may be the reason why U-2 OS cells are unable to activate IRF1 following exposure to A + N. On the other hand, in U-2 OS cells camptothecin stimulates IRF1 regardless of p53 status confirming the conclusions of Tanaka et al. [27]. Thus, IRF1 can be upregulated by at least 3 factors: interferon gamma [38], DNA damaging agents (Figure 10e, [27]) and strongly activated p53 (Figure 10). Thus, upregulating IRF1 is another instrument for p53 to act as an antiviral molecule because IRF1 can activate several genes for antiviral proteins [37].

**Figure 13:**
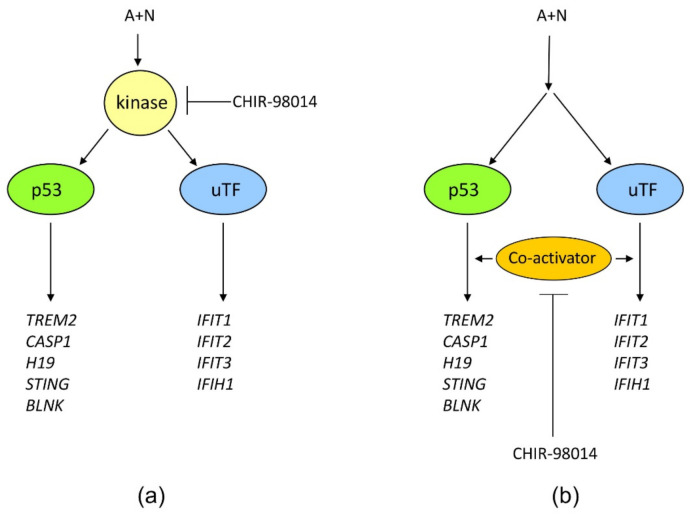
A schematic representation of the two hypotheses (**a**,**b**) generated by the data presented in this paper. There are two groups of innate immunity genes activated by A + N − one group is stimulated by p53 and the other is activated independently from p53. The other group must be under the control of a transcription factor, which is currently unidentified (uTF). The outcome of activity of both transcription regulators (p53 and uTF) is modulated by a kinase inhibited by CHIR-98014. This kinase plays crucial role in gene regulation in stress conditions elicited by A + N. In principle, the kinase can act upstream (**a**) or downstream (**b**) from p53 or uTF.

The *IFIT1* and *IFIT3* genes can be stimulated by IRF1-they contain IRF1 binding sites detected by ChIP-Seq method in monocytes [26]. We observed that IFIT1 and IFIT3 proteins are strongly stimulated by A + N and by camptothecin. Using our earlier model, we did not find convincing evidence that p53 participates in their upregulation [4]. Using A549 cells with p53 expression knocked down by CRISPR/Cas9 method we noticed that p53 can participate in their activation (Figure 11b). In principle, p53 can do it directly or indirectly, through stimulation of IRF1. The detailed analysis of IRF1 target genes in lung cancer cells will be the subject of another, more extended study. Because the involvement of p53 in regulation of IFIT1 and IFIT3 was not clear-cut we decided to use another model. We selected clones of cells with the knockout of p53. We found two clones with no detectable p53. The cells did not activate gene for p21 protein, which is archetypal p53-regulated gene. Yet, these two clones were able to activate IFIT1, IFIT3 as well as IFIH1-another innate immunity gene selected through transcriptomic analysis. Moreover, the p53-null cell line NCI-H1299 also was able to activate IFIT1 and IFIT3 (Figure 12). Thus, definitely, these two genes can be activated without assistance from p53 in cells exposed to A + N. So can be other innate immunity genes, which we picked out from transcriptomic analysis-IFIT2 and IFIH1. The expression of these four genes can be blocked or strongly attenuated by CHIR. Thus, surprisingly, this inhibitor can also attenuate the expression of the genes, which are not regulated by p53. Based on our results, we created two models explaining some of our observations (Figure 13). In principle, they are not mutually exclusive. Treatment of cells with A + N triggers a signaling system containing an unidentified kinase, which is sensitive to the inhibitory activity of CHIR. This kinase can activate by phosphorylation an unidentified transcription factor, which stimulates the expression of IFIT1, IFIT2, IFIT3 and IFIH1 independently from p53. This kinase can also phosphorylate p53. The p53 as a transcription factor is not an on/off switch but its activity can be modulated by the number and type of posttranslational modifications. Thus, by getting a new modification, p53 can acquire the ability to interact with additional gene regulatory DNA sequences what enables the activation of new set of genes. Alternatively, new modification may enable p53 to interact with new transcriptional co-activator. Regardless the mechanism, additional modification can expand the set of regulated genes and/or it may increase the degree of gene activation [14]. We suspect that the kinase inhibited by CHIR does to p53 what was hinted above. It modifies p53 in a way that allows for strong activation of some (e.g., *TREM2*, *BLNK*, *TMEM173*, *H19*) but no other genes (*APOE*, *FAS*, *PMAIP*, *BBC3*) (Figure 2 and Figure 7). The Venn diagram shows (Figure 1) that about half of genes upregulated by A + N is inhibited by CHIR to some degree. The genes very strongly influenced by CHIR can be seen on heatmaps (Figure 2).

In the alternative scenario, the kinase inhibited by CHIR can act downstream from both transcription factors functioning as a common co-activator of both p53-regulated genes and the genes regulated by the other transcription factor.

In our opinion the most intriguing conclusion of our work is that there is a kinase, sensitive to CHIR, which is indispensable for activation of many innate immunity genes regulated by p53 and some other transcription factor. Moreover, this kinase has anti-apoptotic activity because its inhibition promotes apoptosis in several cell lines. Its identification will have the impact on our understanding of the interplay between cancer and immune cells.

## 4. Materials and Methods

### 4.1. Cell Culture, Reagents and Treatment

A549 (lung adenocarcinoma, American Type Culture Collection (ATCC, Manassas, VA, USA), NCI-H292 (lung cancer, ATCC), and U-2 OS (osteosarcoma, ATCC) cells were grown as previously described [2]. NCI-H460 (lung cancer, ATCC) cells were grown at 37 °C/5% CO2 in RPMI-1640 supplemented with 2 mM L-glutamine, 4.5 g/L glucose and 1 mM sodium pyruvate, supplemented with 10% heat-inactivated fetal bovine serum (FBS; Invitrogen, Carlsbad, CA, USA) and 1% penicillin–streptomycin (Sigma-Aldrich, St. Louis, MO, USA).

The stock solutions of chemicals were prepared in DMSO: actinomycin D (10 μM; Sigma-Aldrich, St. Louis, MO, USA), camptothecin (10 mM; Calbiochem-Merck, Darmstadt, Germany), nutlin-3a (10 mM; Selleck Chemicals LLC, Houston, TX, USA), CHIR-98014 (5 mM, Selleck Chemicals, Houston, TX, USA), PHA-793887 (10 mM, Selleck Chemicals, Houston, TX, USA). Stock solutions were diluted in culture medium to the following concentrations: 5 nM actinomycin D, 5 μM nutlin-3a, 5 μM camptothecin. CHIR-98014 and PHA-793887 were diluted to concentrations indicated in the Results section. Control cells were mock-treated with medium containing DMSO.

The apoptotic cells were analyzed using PE Annexin V Apoptosis Detection Kit I (BD Biosciences, San Jose, CA, USA) according to manufacturer’s protocol using BD FACSCanto™ cytometer (Becton Dickinson, San Jose, CA, USA). The data were analyzed using BD FACSDiva™ (Becton Dickinson, San Jose, CA, USA) software.

### 4.2. The Knock-Down of Gene Expression

CRISPR Double Nickase Plasmids were used to disrupt expression of either *TP53*, *GSK3A* or *GSK3B* genes (Santa Cruz Biotechnology, Dallas, TX, USA). A set targeting a gene contains a pair of plasmids, not purified gRNAs and Cas9 nuclease. Each of the pair of plasmids encodes modified Cas9 nuclease and a unique, target-specific guide RNA (gRNA). Thus, these molecules are produced from plasmids in successfully transfected cells. Each gRNA targets a sequence on complementary DNA strand. The target sequences are offset by approximately 20 bp to allow for gene knockout with relatively high specificity. Following transfection, the Cas9 nuclease molecules guided by gRNAs on both DNA strands generate a staggered cut in target gene. This double-strand break is repaired in error prone fashion generating either frameshift mutations or amino acid deletions. The plasmids with the following catalogue numbers were employed: sc-416469-NIC for *TP53*, sc-400518-NIC for *GSK3A*, sc-400090-NIC for *GSK3B*. As a control we used Control Double Nickase Plasmid (sc-437281). For the transfection we employed a procedure suggested by the vendor. In short, cells were plated in the wells of the 6-well plate and after overnight culture in antibiotic-free medium, they were transfected with the plasmids. In order to get p53-deficient U-2 OS cells, they were transfected with UltraCruz Transfection reagent (Santa Cruz Biotechnology)-1 µg of DNA, 5 µL of the transfection reagent per well. In order to get p53-deficient or GSK-3-deficient A549 cells, they were transfected with FuGENE 6 transfection reagent (Promega, Madison, WI, USA)-2 µg of DNA, 6 µL of FuGENE 6). Following transfection the plasmids produced Cas9 nuclease, two guide RNA sequences and the puromycin resistance protein coded by one of the plasmids. Seventy two hours after start of transfection, puromycin was added to the medium. The selection with puromycin lasted for 72 h. The purpose of selection was to eliminate cells, which were not successfully transfected, hence expressed neither nuclease nor guide gRNAs able to disrupt expression of target genes. The selection was ended because transient transfection did not generate permanent puromycin resistance. After the transfection and puromycin selection most cells died because transfection is not 100% effective and most of the cells did not acquired plasmid with puromycin-resistance gene. However, the clones that regrew could be expanded and divided normally. At the end of selection, we had individual surviving cells, which could be expected to have the loss of function mutations in one or both copies of target genes. We incubated the cells in fresh, puromycin-free medium in order to expand the culture, what allowed for protein expression studies.

To select p53 knockout clones (or controls for knockout), the cells from the procedure described above were counted and seeded to round-bottom 96-well plate at the calculated density of 0.3 cells per well-hence we could expect to have one cell in every 3 wells. After 10–14 days of culture, the cell populations from individual wells were transferred to larger culture dishes, expanded and tested for p53 expression. The clones expressing no detectable p53 after exposure to A + N were selected for further analyses.

To perform a double-knockdown of *GSK3A* and *GSK3B* in A549 cells, we selected cells with the knockdown of *GSK3B* (or controls for knockdown) performed using CRISPR/Cas9 technology and we transduced them with lentiviruses expressing shRNA molecules directed against *GSK3A* mRNA (or with control lentiviruses). The lentiviruses were purchased from Santa Cruz Biotechnology (cat no. sc-29339 for anti-GSK3A lentivirus and no. sc-108080 for control lentivirus). The procedure was performed according to the manufacturer’s protocol. When the cell cultures were expanded, we assessed expression of both isoforms of GSK-3 by Western blotting.

### 4.3. Semi-Quantitative Real-Time RT-PCR

Total RNA samples were isolated from cells using the RNeasy mini kit (Qiagen, Hilden, Germany). The cDNA was synthesized with MuLV reverse transcriptase and random hexamers (Applied Biosystems, Foster City, CA, USA). Measurements of mRNA levels were performed using Real-Time 2 × PCR Master Mix SYBR (A&A Biotechnology, Gdynia, Poland). The sequence of primers used for RT-PCR are given in Table S1. The primers for β-actin (internal reference gene) were: 5′-GCA AGC AGG AGT ATG ACG AG and 5’-CAA ATA AAG CCA TGC CAA TC (BioTeZ, Berlin, Germany). Amplification was performed on a CFX96 Real-Time System (Bio-Rad, Hercules, CA, USA). In each PCR run, cDNA samples were amplified in triplicate. Relative quantitation of mRNA was carried out using the ΔΔCT method with β-actin as a reference. Mean and standard deviation were calculated from three biological replicates.

### 4.4. Western Blotting

Whole-cell lysates were prepared using IP buffer, supplemented with protease and phosphatase inhibitors as described previously [4]. Aliquots of lysates (35–50 µg) were separated by SDS-PAGE on 8% or 13% gels and electro-transferred onto PVDF membranes. Before incubation with primary antibody, the membranes were incubated for 1 h at room temperature in blocking solution (5% skim milk in PBS with 0.1% Tween-20). The following primary antibodies were obtained from Cell Signaling Technology (Danvers, MA, USA): anti-phospho-Ser33, anti–phospho-Ser37 p53, anti-phospho-Ser46, anti-phospho-Ser392, anti-phospho-GSK-3α (Ser21)(D1G2), anti-phospho-GSK-3β (Ser9)(5B3), anti-BLNK (D3P2H), anti-IFIT1 (D2X9Z), anti-STING (D2P2F), anti-IFI16 (D8B5T), anti-IRF1 (D5E4), anti-MDA5 (IFIH1) (D74E4), anti-TREM2 (D8I4C) anti-caspase-8 (1C12), anti-caspase-9 (rabbit polyclonal), anti-active caspase-3 (Asp 175)(5A1E). Anti-IFIT3 antibody (ab95989), anti-CASP1 antibody (ab179515) were from Abcam (Cambridge, UK). Anti-p53 (DO-1), anti-p21^WAF1^ (F-5), anti-GSK-3α/β (0011-A) and loading control anti-HSC70 (B-6) antibodies were obtained from Santa Cruz Biotechnology. All incubations with primary antibodies were performed overnight at 4 °C in blocking solution. HRP-conjugated secondary antibodies (anti-mouse, anti-rabbit) were detected by chemiluminescence (SuperSignal West Pico or SuperSignal West Femto Chemiluminescent substrate, Thermo Fisher Scientific, Waltham, MA, USA).

### 4.5. Molecular Cloning, Site-Directed Mutagenesis and Luciferase Reporter Assay

The promoter region of BLNK was cloned into the pGL3-Basic reporter vector, which encodes firefly luciferase (Promega, Madison, WI, USA). The human BLNK promoter was amplified by PCR from a genomic DNA sample (A549 cells) using primers: 5′- TTTT GAGCTC TGT TAC CAC CAT GCC ACTG and 5′- TTTT ACGCGT CAG CAT GGT AAG CCT CTG GT. The primers were designed to contain the restriction sites (underlined) for *Sac*I and *Mlu*I, respectively. Amplified DNA was ligated into the respective sites of pGL3-Basic. PCRs was performed with PfuPlus! DNA polymerase mix (EURx, Gdańsk, Poland) to ensure high fidelity DNA amplification. The inserted DNA was sequenced to ensure that the clone contained no mutations.

The mutations of CWWG (W-A or T) sequence in the putative p53 response element (RE) from BLNK promoter were created using GeneArt Site-Directed Mutagenesis PLUS kit (Life Technologies, Carlsbad, CA, USA) with forward (5′ GAC TAT GTG CTC GGG GCGT TTT TAC TTC TCC CTA 3’) and complementary reverse (5′ TAG GGA GAA GTA AAA ACGC CCC GAG CAC ATA GTC 3’) primers (the sites of mutation are underlined).

The luciferase reporter assay was performed as described recently [4]. In short, U-2 OS cells were co-transfected using FuGENE 6 (Promega) with a combination of reporter vector, encoding firefly luciferase under the control of BLNK promoter (wild type or mutant), and expression vector pC53-SN3, encoding wild-type p53 or pC53-SCX3 encoding Val143Ala p53 mutant (a gift from Dr. Bert Vogelstein and Dr. Kenneth W. Kinzler from Johns Hopkins University, Baltimore, MD, USA) [39]. As a negative control, the p53 plasmid was replaced by empty vector. The transfection mixture also contained pRL-TK, encoding Renilla sp. luciferase under the control of HSV-TK promoter (internal control). The next day, the cells were washed with culture medium and incubated with fresh medium for an additional 24 h. The cells were lysed with PLB buffer from the Dual Luciferase Reporter Assay system (Promega, Madison, WI, USA) and the activities of the luciferases were measured. Firefly luciferase activity was normalized against Renilla sp. luciferase activity. Each transfection was performed in triplicate in three independent experiments.

### 4.6. RNA-Seq and the Analysis of Results

A549 cells were treated for 30 h with 5 nM actinomycin D and 5 μM nutlin-3a (A + N), with A + N in the presence of 1 μM CHIR-98014 or with 1 μM CHIR-98014 acting alone. Control cells were mock-treated with culture medium containing solvent (DMSO). At the end of treatment cells were harvested by trypsinization. After washing in PBS, the cell pellets were frozen in −70 °C. Total RNA samples were prepared using the RNeasy mini kit with on-column digestion with DNAse (Qiagen, Hilden, Germany). The quality of RNA samples was determined using an Agilent Bioanalyzer (Agilent Technologies, Santa Clara, CA, USA). High quality RNA samples were prepared (OD 260/280 ratio ≥ 1.8, OD 260/230 ≥ 1.7, RNA integrity number, RIN ≥ 9.0). The RNA sequencing and expression analysis were outsourced to Eurofins Genomics Europe Sequencing (Constance, Germany). Sequencing using Genome Sequencer Illumina HiSeq4000 was performed on random primed cDNA library synthesized from poly-A containing mRNA molecules. Fifty-bp, single-end reads were performed. We had the following number of quality control clean reads: control-65,496,040, A + N-80,592,823, A + N + CHIR-63,207,428, CHIR-68,662,962. Clean reads constituted at least 99.6% of total reads. Quality filtered reads were aligned to the reference human genome (hg19). At least 98.6% of clean reads could be successfully mapped. RNA-Seq reads were aligned to the reference genome using Bowtie aligner (version 2.3.3.1) [40]. TopHat (version 2.0.14) was used to identify the potential exon-exon splice junctions of the initial alignment [41]. Subsequently, Cufflinks assembler and abundance estimation algorithms were employed to identify and quantify the transcripts from the preprocessed RNA-Seq alignment-assembly. After this, Cuffmerge algorithm merged the identified transcript pieces to full length transcripts and annotated the transcripts to the genome annotations (Gencode v27, Ensembl 90). Finally, merged transcripts from treated and control samples were compared using Cuffdiff utility to determine the differential expression levels of given transcripts including the measure of statistical significance of the differences (the *p*-value of the test statistic and the False Discovery Rate-adjusted *p*-value of the test statistic). This analysis was performed using open-source programs published by Trapnell et al. [42].

List of significantly up-regulated and down-regulated genes was created only from transcripts with annotated gene symbols and refseq ID with mean expression > 1 FPKM. In order to avoid dividing by zero for log2FC (fold change) calculations, undetectable expression was transformed into half on minimal detected expression value. Upregulated genes were considered those with log2FC > 1 and *p* value < 0.05 and down-regulated with log2FC < −1 and *p* value < 0.05. Due to exploratory nature of our RNA-seq experiment and further validation of selected gene expression, we used unadjusted *p* value threshold for gene selection.

For functional analysis of significantly up- and down-regulated genes we used g: Profiler tool [43]. Top overrepresented pathways with adjusted *p* value < 0.05, size < 200 and intersection to query size ratio > 0.03 were shown in the Figure 1. All results of functional analysis are shown in Supplementary Tables S5–S10.

## Data Availability

The sequencing files (fastq) are available at Sequence Read Archive under the accession number PRJNA757776. Other, processed sequencing data are available from the first or corresponding author upon request.

## Supplementary Materials

The following are available online at https://www.mdpi.com/article/10.3390/ijms222011072/s1.
