# Supplementary material for: Transcriptome Analysis of Cells Exposed to Actinomycin D and Nutlin-3a Reveals New Candidate p53-Target Genes and Indicates That CHIR-98014 Is an Important Inhibitor of p53 Activity
Source: Int J Mol Sci. 2021 Oct 14;22(20):11072. doi: 10.3390/ijms222011072 (PMC8538697; doi:10.3390/ijms222011072)
Supplement: Supplementary file 1 [file ijms-22-11072-s001.zip › Table S1.pdf]

Table S1. The sequences of RT-PCR primers.

Gene	Sequence 5'->3'	Company
CASP1	TCG CTT TCT GCT CTT CCA CA and TCC ACA TCA CAG GAA CAG GC	Genomed, Warsaw, Poland
STING	TAC ATC GGA TAT CTG CGG CT and TGG GGC AGT TTA TCC AGG AA	Genomed, Warsaw, Poland
H19	AAT CGG CTC TGG AAG GTG AAG and GCT GCT GTT CCG ATG GTG TC	Genomed, Warsaw, Poland
P21(CDKN1A)	CTG CCT CCT CCC AAC TCA and CCC GTG AGC GAT GGA ACT	BioTeZ, BerlinBuch, Germany
BBC3	GGG TGA GAC CCA GTA AGG AT and AGC TTT CCA TTC CGT TTC TT	RealTimePrimers.com, Elkins Park, PA, USA
DRAM1	AGA CTC CAT CTT TTC ACC CAA A and GCT CTT CAC CTT TCA AGC CTA A	BioTeZ, BerlinBuch, Germany
APOE	AAGCGA CCCAGT GCCAGT TC and GTCCTT CCCCAG GAGCCG AC	Genomed, Warsaw, Poland
BLNK	TCTGCTGAAGAGGCATTGCAC and GTTTGGAATCATGGCCAGAGC	Genomed, Warsaw, Poland
CRYAB	GCACTT CTCCCC AGAGGA AC and CCATTC ACAGTG AGGACC CC	Genomed, Warsaw, Poland
CCNG2	TTGATT GAGGCT ACCCCG GA and AGAACA GACTCC AATGCA AGACA	Genomed, Warsaw, Poland
FAS	TGGGGA TGAACC AGACTG CG and CTGGGT CCGGGT GCAGTT TA	Genomed, Warsaw, Poland
PMAIP	CCGAAG ATTACC GCTGGC CT and ACAATG TGCTGA GTTGGC ACTG	Genomed, Warsaw, Poland
GDF15	TCAAGG TCGTGG GACGTG AC and TGCGGA CGAAGA TTCTGC CA	Genomed, Warsaw, Poland
BCL2L15	TGCCAC AGGATG CAGGAA GT and AGGTCC ACAAGT GGTGTG TGA	Genomed, Warsaw, Poland
IRF1	AAAGTC GAAGTC CAGCCG AG and TGTTGT AGCTGG AGTCAG GG	Genomed, Warsaw, Poland
IFIT2	AGGAAGGGTGGACACGGTTA and TGCCTCAGAGGGTCAATGGC	Genomed, Warsaw, Poland
