# Supplementary figures and images for: Transcriptome Analysis of Cells Exposed to Actinomycin D and Nutlin-3a Reveals New Candidate p53-Target Genes and Indicates That CHIR-98014 Is an Important Inhibitor of p53 Activity
Source: Int J Mol Sci. 2021 Oct 14;22(20):11072. doi: 10.3390/ijms222011072 (PMC8538697; doi:10.3390/ijms222011072)

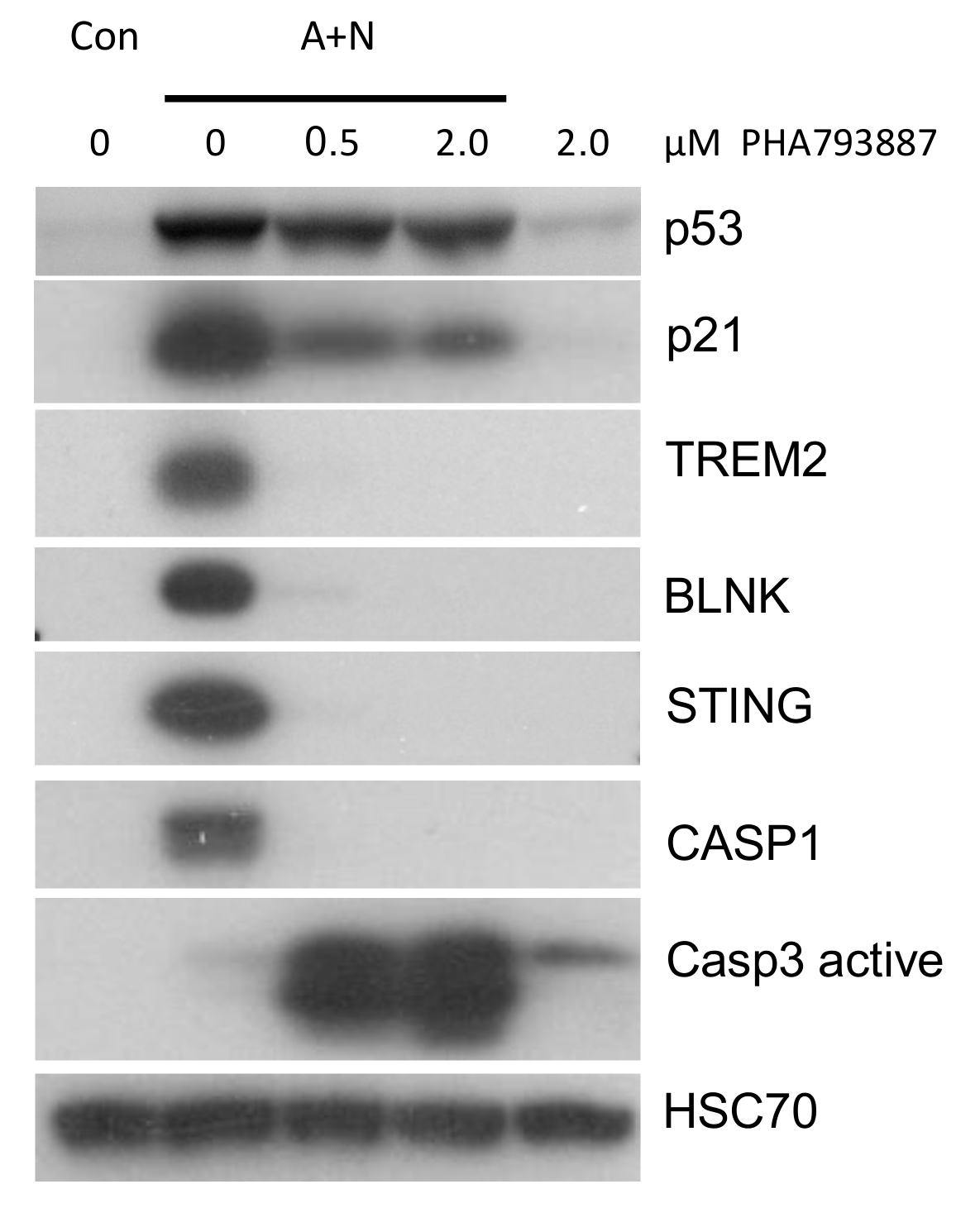

Supplement: Supplementary file 1 [file ijms-22-11072-s001.zip › Fig S1.tif]
